# Supplementary material for: A machine learning approach to quantify the specificity of colour–emotion associations and their cultural differences
Source: R Soc Open Sci. 2019 Sep 25;6(9):190741. doi: 10.1098/rsos.190741 (PMC6774957; doi:10.1098/rsos.190741)
Supplement: Description of the data set for the manuscript "A machine learning approach to quantifying the specificity of color-emotion associations and their cultural differences" [file rsos190741supp2.pdf]

Description of the data set for the manuscript "**A machine learning approach to quantify the specificity of color-emotion associations and their cultural differences"**, Royal Society Open Science, by Domicele Jonauskaite, Jörg Wicker, Christine Mohr, Nele Dael, Jelena Havelka, Marietta Papadatou-Pastou, Meng Zhang, and Daniel Oberfeld (oberfeld@uni-mainz.de)

Each row represents the ratings of the intensity of 20 emotions associated with the color term given by variable "color", by participant identified by variable "subject"

Variables:

lang: language in which the survey was presented = mother tongue = country of origin. CN = China, DE = Germany, GB = United Kingdom, GR = Greece,

subject: code for the participant

color: color term

admiration to *shame*: ratings of emotion intensity. 0 = no association between the color term and a given emotion. 5 = strongest intensity of an associated emotion.
